# Value-Based Model: A New Perspective in Medical Decision-making

**DOI:** 10.3389/fpubh.2016.00118

**Published:** 2016-06-13

**Authors:** Silvia Riva, Gabriella Pravettoni

**Affiliations:** ^1^Department of Oncology and Onco-Hematology, University of Milan, Milan, Italy; ^2^Applied Research Division for Cognitive and Psychological Science, European Institute of Oncology (IEO), Milan, Italy

**Keywords:** value-based decision-making, medical decision-making, medical decisions, health-related quality of life, patient outcomes

The problem of how to use financial resources beneficially in health care, despite limited budgets, reduced personnel, and inadequate time, goes beyond the realm of health economists (1). How do we use existing resources to prioritize one health program over another? Does a specific program provide a real and measurable improvement in terms of perceived health-related quality of life (HRQOL)? What decisions should we make?

In the recent years, the HRQOL index has become an asset for evaluating patient health status in health programs, therapies, and clinical trials (2). Created as a specific psychological construct for use in health contexts, the HRQOL index is now widely used and evaluated by health economists, epidemiologists, clinicians, and nurses. Today, the notion that health status is not only determined by survival or absence of disease but also closely related to quality of health is universally accepted (3, 4).

Health-related quality of life has been defined as the functional effect of a medical condition and/or treatment on a patient. Thus, HRQOL is a subjective and multidimensional parameter that encompasses physical and occupational functioning, psychological state, social interaction, and somatic sensation (5). Health is perceived by the person as a state that lies beyond the presence of a disease or the severity of a condition. HRQOL is influenced by several factors that include not only psychological and emotional aspects, individual behavior, and attitudes but also personal experiences, culture, and religious beliefs (6, 7). Owing to this subjectivity, patients with the same medical condition may have very different perceptions of HRQOL. For example, patients with some hematological conditions, such as hemophilia, may experience a good HRQOL despite having several disease-related complications, whereas others with the same disorder but better clinical conditions may perceive a poor HRQOL (8–12). Similar results have been obtained in patients with other chronic conditions, such as back pain (13, 14).

Evaluation of HRQOL has been encouraged over the last two decades to obtain a broader perspective of health status in patients and to identify which therapeutic choices and programs to pursue (15). Health economists may use the HRQOL construct as a deciding factor in applying economic analyses. For example, in cost-benefit, cost-effectiveness, or cost-utility analyses, HRQOL may represent one indicator for measuring *effectivenes*. (e.g., success of a health program, specific treatment, or regulatory policy) (16). In this context, effectiveness may be measured as a final outcome, such as the number of life-years gained or number of lives saved, by applying a certain program or proposing a certain treatment. Alternatively, it may be measured as an intermediate outcome in a health program, such as the number of days lost from school or work, perceived HRQOL, treatment satisfaction, or psycho-emotional well-being (17).

The HRQOL construct focuses on the patient’s perceived state of health as a reference value in evaluating programs and making medical decisions. In this light, we wish to discuss the potential of the HRQOL and psychological science in a new medical paradigm that has been recently described as an alternative approach to the traditional paradigm of evidence-based medicine, namely, the value-based medicine approach.

## Psychological Perspective of the Value-Based System

Health systems are increasingly being burdened by enormous public expenditures, and health policy decisions made to contain costs have so far been ineffective. A new direction for facilitating health-care management and containing health-care costs may be represented by Porter’s theoretical value-based model (18, 19). According to this model, choices in the health context should be oriented toward “adding value for the patient.” Health systems may support interventions and choices that appear expensive in the short term but provide better outcomes in the long term, with a drastic reduction of costs and public expenditures (20).

The value-based model is different from the evidence model, but they are linked together. While the traditional evidence-based medicine often measures the improvement gained in length of survivorship, but generally does not measure the significance in terms of quality-of-life improvement, the value-based medicine integrates the best results of evidence-based medicine (it uses best evidence-based data) to a superior level of analysis by combining the HRQOL evaluations of patients “with a disease in concerning the value of an intervention” (20).

According to this perspective, it is necessary to promote integrated interventions that care for the whole patient, with the psychological aspects of care taking on increasing importance (21, 22). The value-based model highlights the importance of health outcomes, including HRQOL and patient well-being, in making appropriate medical decisions. Sustainable and improved health-care management and costs may be obtained by measuring concrete patient health outcomes (patient utility) and abandoning evaluations of general health structure costs, which had been proposed by the traditional evidence-based medicine model (20). The value-based model proposes that health outcomes be measured over a longer period, and that the patient’s health status be compared in a comprehensive and integrated manner that considers all aspects of the patient’s life (i.e., physical health, psychological aspects, and social life). Thus, the term value cannot be reduced to the cost of an intervention but must include a general evaluation of the patient’s overall well-being. Although this general evaluation includes HRQOL, the construct must be evaluated in a wider perspective together with all other health outcomes (e.g., utility, satisfaction, patient preferences, and level of functioning). According to Porter, health outcomes should be measured by medical condition (e.g., cancer), not by specialty (e.g., oncology) or intervention (e.g., chemotherapy). More importantly, health outcomes should be considered across the full period of care for the condition and should track the patient’s health status after conclusion of care (23).

In practice, the consideration of health outcomes, including HRQOL, represents only one tier for the development of an effective valued-based system in health care. Other tiers may include the development of (1) integrated health-care practice units comprising clinical and non-clinical health-care team members; (2) bundled payment systems that cover the full care cycle over a defined period of time or for a defined population, based on improvement of health outcomes; (3) integrated care delivery systems able to eliminate fragmentation of care; and (4) local network opportunities among clinics and medical institutions (24). All of these practical proposals may favor a change from the evidence-based model (centered purely on scientific evidence and organized around what physicians do) to a valued-based model (centered on the patient and organized around what patients need) (23, 24). The value-based medicine model seems to comply with the definition of health reported by the World Health Organization (WHO): “Health is a state of complete physical, mental, and social well-being and not merely the absence of disease or infirmity” (25).

Health outcomes measure the effects of treatment and care that patients receive. Outcomes include, for example, remission of the condition, degree of patient functionality, recovery status, and emotional well-being. The value-based medicine model highlights the importance of the psycho-social dimensions, which enrich the traditional clinical picture by describing the patient not only as sick but also a person with a specific biophysical and psychological profile who is inserted in a social context (family, work, etc.). The direction proposed by value-based medicine indicates that hospitals need to place the patient in the center, evaluating and integrating all human and social aspects of a person.

According to this model, outcome indicators need to be appropriate and specific for each particular condition. They should not be measured for a single event or over a short time frame but rather should be measured throughout the entire course of treatment (e.g., inpatient, outpatient, and day hospital phases). This perspective advocates the importance of a personalized approach to medicine, particularly the Five Ps of Personalization, which includes a Predictive, Precise, Preventive, Participatory Medicine that is sensitive to Psychological aspects and the patient’s cognitive characteristics (26). In particular, consideration of the psychological aspects requires an investigation of the psycho-cognitive variables that come into play when each individual makes a personal choice (27–29) with respect to the health, treatment, and care pathways.

## Author Contributions

SR conceived the work, reviewed literature, and wrote the manuscript. GP supervised the work and wrote the manuscript. All the authors have agreed on the final version of the manuscript.

## Conflict of Interest Statement

The authors declare that the research was conducted in the absence of any commercial or financial relationships that could be construed as a potential conflict of interest.
